# Extremely high-performance visible light photodetector in the Sb_2_SeTe_2_ nanoflake

**DOI:** 10.1038/srep45413

**Published:** 2017-03-28

**Authors:** Shiu-Ming Huang, Shih-Jhe Huang, You-Jhih Yan, Shih-Hsun Yu, Mitch Chou, Hung-Wei Yang, Yu-Shin Chang, Ruei-San Chen

**Affiliations:** 1 Department of Physics, National Sun Yat-Sen University, Kaohsiung 80424, Taiwan; 2 Department of Materials and Optoelectronic Science, National Sun Yat-Sen University, Kaohsiung 80424, Taiwan; 3 Taiwan Consortium of Emergent Crystalline Materials, TCECM, National Sun Yat-Sen University, Kaohsiung 80424, Taiwan; 4 Graduate Institute of Applied Science and Technology, National Taiwan University of Science and Technology, Taipei 10607, Taiwan

## Abstract

The photocurrent was performed in the Sb_2_SeTe_2_ topological insulator at a wavelength of 532 nm. It exhibits extremely high performance that the responsivity and the photoconductive gain reach 2293 AW^−1^ and 5344 at 1 V. This high photoresponse is orders of magnitude higher than most reported values in topological insulators and two-dimensional transitional metal dichalcogenides. This finding suggests that the Sb_2_SeTe_2_ nanoflake has great potential for future optoelectronic device applications.

A system that generates a high photocurrent in response to light may be used as a photosensor. The light penetration distance is very short; thus, the photoresponse properties are dominated by the carriers near the material surface. A material with a relatively high surface carrier dominance can be expected to perform as a relatively efficient photodetector. To optimize the photoresponse, various types of nanostructured materials, with high surface-to-volume ratios and high levels of photoresponse, were investigated^1^,^2^,^3^,^4^,^5^,^6^. Recently, two-dimensional materials such as graphene^7^,^8^, graphene-based heterostructures^1^,^2^,^3^,^4^, and two-dimensional transitional metal dichalcogenides (TMDs) have attracted noteworthy attention^9^,^10^,^11^,^12^,^13^,^14^,^15^,^16^. These two-dimensional materials demonstrate excellent photoelectrical performance because they have high surface-to-volume ratios and abundant surface carriers.

Three-dimensional topological insulators are promising materials because they offer insulating bulk states and a gapless conducting surface state. These insulators have a surface state that is topologically protected by a time reversal symmetry, which is induced by a strong spin-orbit interaction. This remarkable surface state has garnered intensive theoretical and experimental attention and had been a recent research topic^17^,^18^. The linear dispersions in the surface state and the extremely high carrier mobility levels make these insulators promising candidates for optical electrical devices^19^,^20^. The photoelectrical characteristics of the Bi-based topological insulators have been investigated and have revealed promising responses^21^,^22^. It is reported that the Bi_2_Te_3_ topological insulator based heterostructures^1^,^23^,^24^ and PLD-grown Bi films^25^ reveal ultrahigh responsivity in wide wave range. Recently, it was reported that Sb_2_Te_3_ thin films offer higher photoelectrical responses than that in Bi-based topological insulators^26^.

In this paper, we report on the photocurrent produced by a 532-nm wavelength in a Sb_2_SeTe_2_ topological insulator. The experimental results reveal extremely high performance; specifically, the responsivity and the photoconductive gain reached 2293 AW^−1^ and 5344 at a bias of 1 V. These observations are orders of magnitude higher than most reported values in other topological insulators and two-dimensional TMDs, which suggests that Sb_2_SeTe_2_ nanoflakes have great potential for future optoelectronic device applications.

## Experimental method

Single crystals of Sb_2_SeTe_2_ were grown by a homemade resistance-heated floating zone furnace (RHFZ). The starting raw materials of Sb_2_SeTe_2_ were mixed according to the stoichiometric ratio. At first, the stoichiometric mixtures of high purity elements Sb (99.995%), Se (99.995%) and Te (99.995%) were melted at temperatures of 700 ~800 °C for 20 h, and then slowly cooled to room temperature in an evacuated quartz glass tube. The resulting material was used as a feeding rod for the following RHFZ experiment. After growth, the crystals were then furnace cooled to room temperature. The as-grown crystals were cleaved along the basal plane, producing a silvery shining mirror-like surface, and then prepared for the further experiments. The Raman^27^, EDS and XPS^28^ spectrum support that the crystal is Sb_2_SeTe_2_.

**Figure 1:**
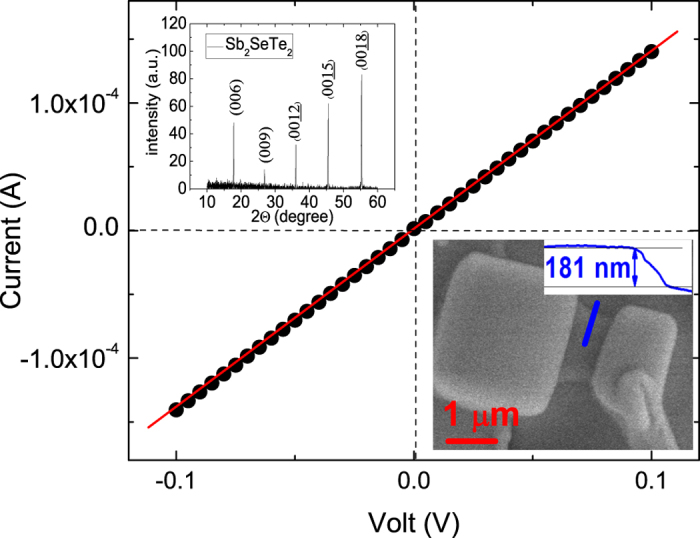
Left-top inset shows the XRD of the Sb_2_SeTe_2_. The sharp peaks indicate the high crystallinity of the Sb_2_SeTe_2_ crystal. The right-bottom inset shows a SEM picture of the Sb_2_SeTe_2_ nanoflake. The red line is the scale bar of SEM. The blue curve is the AFM thickness profile and the sample thickness is 181 nm. Two Pt contacts were deposited on the nanoflake to measure the photocurrent. The linear current-voltage curve indicates the ohmic contact between the Pt electrodes and the Sb_2_SeTe_2_ nanoflake.

**Figure 2:**
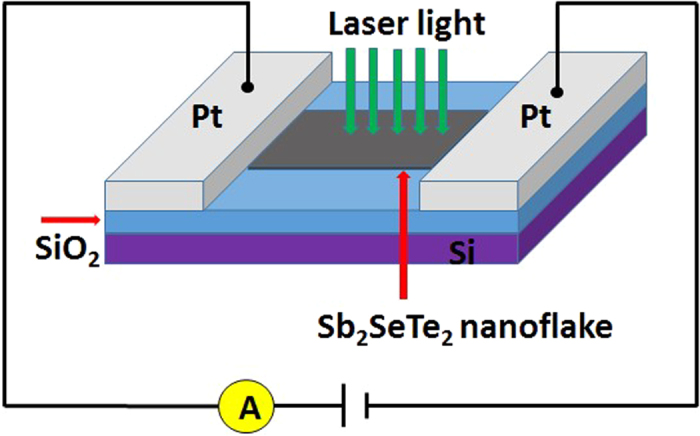
Schematic of the Sb_2_SeTe_2_ nanoflake device illustrating the photoelectrical measurement setup and the light that illuminated it. The wavelength of the light was 532 nm.

The Sb_2_SeTe_2_ nanoflakes were obtained by exfoliating bulk crystals using dicing tape and were then dispersed on the insulating SiO_2_ (300 nm)/*n*-Si templates with pre-patterned Ti/Au circuits. Two platinum (Pt) metal contacts were subsequently deposited on the selected Sb_2_SeTe_2_ nanoflakes using focused-ion beam (FIB) technique (shown in the right-bottom inset of Fig. 1). The thickness of a nanoflake is determined by the atomic force microscopy; here, the nanoflake was 181-nm thick, 708-nm long, and 1667-nm wide. The current-voltage characteristic reveals a linear dependence that indicates the ohmic contacts in the sample; the conductivity was approximately 33.7 *S*/*cm*. The left-top inset within Fig. 1 shows the X-ray diffraction of the Sb_2_SeTe_2_; the sharp peaks indicate that the Sb_2_SeTe_2_ crystal has high crystallinity. Our previous works show that the physical parameters extracted from XPS, Raman spectrum, ARPES and the quantum SdH oscillation are consistent. That supports the Sb_2_SeTe_2_ crystal reveals high quality and uniformity. Figure 2 presents the schematic of the Sb_2_SeTe_2_ nanoflake device, illustrating the photoelectrical measurement setup and the light that illuminated it. The wavelength of the illuminating light was 532 nm.

## Results and Discussion

**Figure 3:**
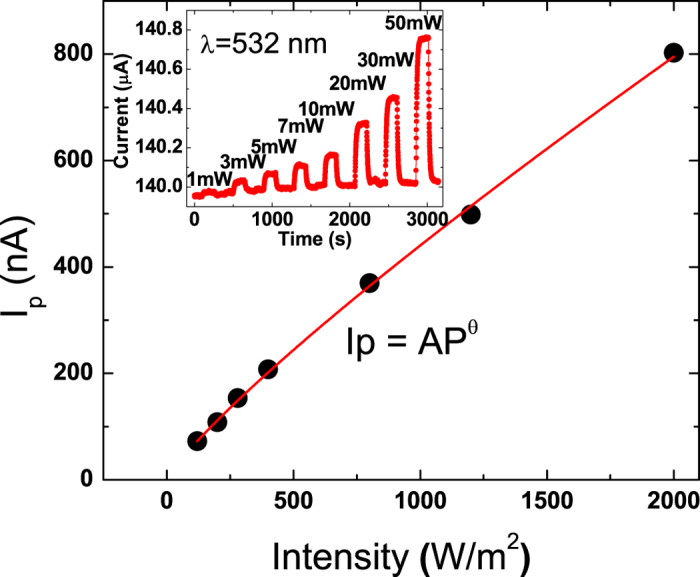
Left-top inset depicts the photocurrents produced by illumination at different power levels. The measured photocurrent is a function of light power intensity, and can be accurately described by a simple power law relation.

The inset of the Fig. 3 shows that the measured current of our Sb_2_SeTe_2_ nanoflake under light illumination with light power that ranges from 1 to 50 mW that is corresponding to the power intensity of 40 to 2000 Wm^−2^. It clearly indicates that the current increases with increasing light power. The overall response time is approximately 10 s; which is shorter than the reported values of Sb_2_Te_3_ films^26^, but longer then the values of Bi-based topological insulators^21^,^22^. Here the photocurrent is presented as a function of the power intensity at bias of 0.1 V (Fig. 3). For quantitative analysis, the relationship between the photocurrent and the light intensity can be fitted to the simple power law relation, *I*_*p*_ = *AP*^*θ*^, where the *A* is a constant for the wavelength of the illuminating light, *P* is the power intensity of the light that illuminates the device, and *θ* is a constant related the photosensitivity of the device. As Fig. 3 reveals, the experimental data agrees with the power law relation and the fitting result gives a *θ* of 0.85.

**Figure 4:**
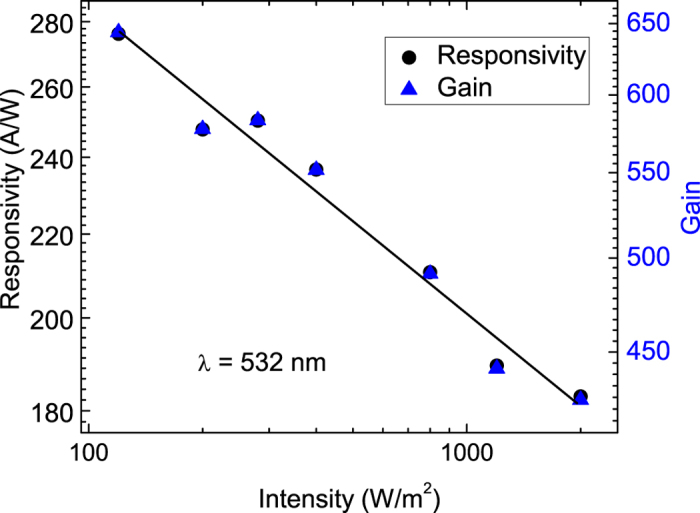
Responsivity and photoconductive gain as functions of the light power intensity at a wavelength of 532 nm. Both responsivity and photoconductive gain increase as the light power intensity decreases.

To quantitatively determine the performance of the Sb_2_SeTe_2_ nanoflake under illumination, responsivity, *R*, and the photoconductive gain, *G*, are calculated through the following equations;


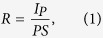



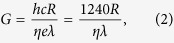


where *I*_*P*_, *P, S, h, c, e, η* and *λ* are the photocurrent, the light intensity, the effective area, Planck’s constant, the velocity of light, the charge of an electron, the quantum efficiency (for convenience, we assume *η* = 1) and the wavelength, respectively. The *G* for a wavelength is proportional to the *R* at the same wavelength. Figure 4 depicts *R* and *G* as functions of the light intensity at a constant bias of 0.1 V, and reveals that the *R* and *G* decrease as the power intensity increases. Specifically, the *R* and *G* are 276 AW^−1^ and 643 at a power intensity of 120 Wm^−2^.

**Figure 5:**
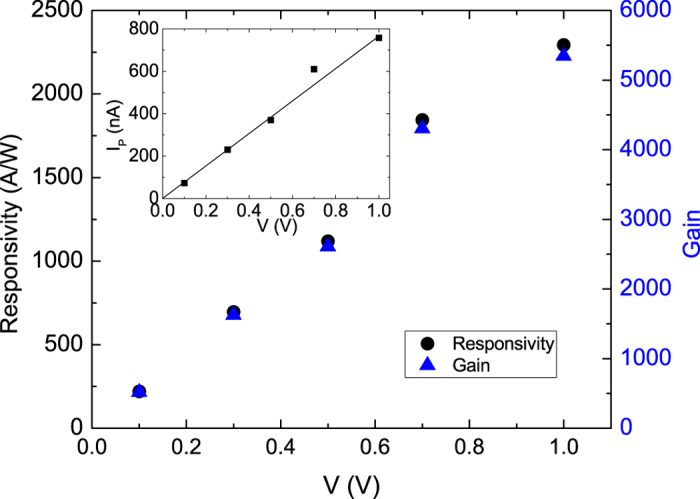
Left-top inset shows the linear relation of a photocurrent to the applied bias at a wavelength of 532 nm and a power intensity of 280 W/*m*^2^. The main figure shows the responsivity and the photoconductive gain as functions of the applied bias at a wavelength of 532 nm and the power intensity of 280 W/*m*^2^.

The photocurrent is strongly related to the applied bias. To comprehensively investigate the intrinsic optoelectronic characteristics in the Sb_2_SeTe_2_ nanoflake, an experiment on bias-dependent photocurrents was performed. As shown in the inset of the Fig. 5, the photocurrent was linearly related to the applied bias; specifically, the observed photocurrent was approximately 0.8 *μ*A at 1 V and a light power intensity of 280 Wm^−2^. This linear bias-dependent increment of the photocurrent can be attributed to the increment drift velocity and the reduced carrier transit time caused by applied bias. Expressed as 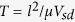, *T* is the carrier transit time, *l* is the device length, *μ* is the carrier mobility, and *V*_*sd*_ is the applied bias. This indicates a system with higher carrier mobility; and, a higher bias might decrease the carrier transit time, and produce a higher photocurrent. Figure 5 also indicates that the evaluated *R* and *G* are functions of bias, to which both linearly relate. At 1 V, The *R* and *G* reach 2293 AW^−1^ and 5344, respectively.

To qualitatively identify the optical performance of the Sb_2_SeTe_2_ nanoflake, the reported values were collected. Table 1 presents a list of the reported *R* and *G* values for topological insulators and two-dimensional TMDs, and clearly reveals that the *R* and *G* values for our Sb_2_SeTe_2_ are orders of magnitude higher than most the reported values in topological insulators and two-dimensional TMDs under similar conditions. That suggests that the Sb_2_SeTe_2_ has the potential to deliver extremely high-performance photocurrent-related applications.

Aside from the high quality of the crystalline sample and the large surface-to-volume ratio, several possible causes might lead to this extremely high photoresponse. First, the photoresponse is extremely sensitive to the condition of sample surface. In addition to the reduction of the effective response area, surface defects and oxidation reduce carrier mobility and life time. One prior study reported that adsorbed molecules on a surface reduce the carrier’s life time; thus, the photoresponse of a material in a vacuum is higher than the photoresponse of the same material in the air^29^. Our previous work revealed that the surface state carrier transport characteristics in our Sb_2_SeTe_2_ topological insulator can tolerant surface oxidation and molecules adsorbed on the sample’s surface; such molecules might come from unavoidable pollution during the fabrication process or from sample transference^28^. Therefore, less effective defective materials might impair the surface electron transport properties of our Sb_2_SeTe_2_ sheet, and the proposed nanoflake might be very effectively by comparison. Second, in addition to the artificial and extrinsic factors, *R* and *G* values are directly related to carrier mobility. The reported *R* and *G* in MoS_2_ and WSe_2_ flakes were positively related to the field-effect carrier mobility^30^. The surface state carrier mobility of our Sb_2_SeTe_2_ topological insulator was approximately 55.5 *cm*^2^*V*^−1^*s*^−1^ at room temperature^31^; that is one order larger than the previously reported value (4 *cm*^2^*V*^−1^*s*) for a single-layer MoS_2_ flake^30^. Third, it is noteworthy that previous works have revealed that graphene-based heterostructure greatly enhances photoresponse because electron have high mobility in graphene and two-dimensional TMDs demonstrate enhanced adsorption ratios. The *R* and *G* values of our Sb_2_SeTe_2_ are orders of magnitude higher than most reported values in topological insulators and two-dimensional TMDs, and are only lower than the reported values in the nanowires^5^,^6^,^32^. and graphene-MoS_2_ hybrid structure^1^. Theoretical calculation shows that the surface state Dirac point lies at the energy gap of the bulk state in Sb_2_SeTe_2_, and our previous work supported that the Fermi level is below the Dirac point. This energy band structure is similar to the graphene-MoS_2_ hybrid structure and might lead to the observed high photoresponse.

Detectivity, that is an important figure-of-merit in evaluating the ability of a photodetector to detect weak signal, is another important indices used to characterize the performance of photodetectors^33^. The specific detectivity (D^*^) is calculated through the relation:


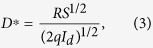


where *R, S, q*, and *I*_*d*_ are the responsivity, effective area of light illumination, electronic charge, and dark current. By using the experimental data, the detectivity is determined to be 4.5 × 10^8^ Jones.

## Conclusion

A photocurrent experiment was performed in a Sb_2_SeTe_2_ topological insulator nanoflake at a wavelength of 532 nm. It exhibited extremely high performance; the responsivity and the photoconductive gain were 2293 AW^−1^ and 5344 at 1 V, respectively. This high photoresponse was orders of magnitude higher than most reported values in topological insulators and two-dimensional TMDs. This finding suggests that the Sb_2_SeTe_2_ nanoflake has remarkable potential for future optoelectronic device applications.

## Additional Information

**How to cite this article**: Huang, S.-M. *et al*. Extremely high-performance visible light photodetector in the Sb_2_SeTe_2_ nanoflake. *Sci. Rep.* **7**, 45413; doi: 10.1038/srep45413 (2017).

**Publisher's note:** Springer Nature remains neutral with regard to jurisdictional claims in published maps and institutional affiliations.

## Figures and Tables

**Table 1:** List of the reported responsivity and gain values of photocurrents in topological insulators and two-dimensional transition metal dichalcogenides.

material	wavelength (nm)	Bias (V)	Responsivity (AW^−1^)	Gain (EQE)	reference
Sb_2_SeTe_2_ nanoflake	532	1	2293	5344	This work
Sb_2_SeTe_2_ nanoflake	532	0.1	276	643	This work
Sb_2_Te_3_ film	980	0.01	0.26	0.33	ref. 26
Sb_2_Te_3_ film	980	0.1	2.31	2.93	ref. 26
Sb_2_Te_3_ film	980	1	21.7	27.4	ref. 26
Bi_2_Se_3_ nanowire	1064	0.1	207	241	ref. 21
Bi_2_Se_3_ nanowire	1064	0.15	300	350	ref. 21
Bi_2_Te_3_ polycrystal	1064	0.3	3 × 10^−5^	3.85 × 10^−5^	ref. 22
graphene – Bi_2_Te_3_	1550	1	0.22	0.17	ref. 34
graphene – Bi_2_Te_3_	980	1	10	11	ref. 34
graphene – Bi_2_Te_3_	532	1	36.7	85.8	ref. 34
Bi_2_Se_3_ nanosheet (exfoliated)	532	0.6	20.4 × 10^−3^		ref. 35
Pristine Bi_2_Se_3_ bulk	532	0.6	2.45 × 10^−3^		ref. 35
Heat-treated Bi_2_Se_3_ nanosheets	532	0.6	16.1 × 10^−3^		ref. 35
Graphene	532	0.1	8.61		ref. 7
Graphene	1550	0.4	6 × 10^−3^		ref. 8
GaSe	254	5	2.8	13.67	ref. 9
GaS	254	2	4.2	20.5	ref. 10
MoS_2_	670	1	4.2 × 10^−4^		ref. 11
MoS_2_	532	5	~6		ref. 12
MoS_2_	532	10	0.57	13.3	ref. 13
MoS_2_	532	1	780	1840	ref. 14
MoS_2_	633	1	120		ref. 15
MoS_2_ nanoflake	532	1	30	103	ref. 29
MoS_2_ nanoflake	561	8	880		ref. 30
MoS_2_	655	5	4.1		ref. 36
APTES-doped MoS_2_	655	5	56.5		ref. 36
OTS-doped MoS_2_	655	5	0.36		ref. 36
WS_2_	655	5	20		ref.^36^
APTES-doped WS_2_	655	5	0.59		ref. 36
OTS-doped WS_2_	655	5	36.4		ref. 36
WS_2_ film	635	9	0.7	137%	ref.^37^
WSe_2_ film	635	10	0.92	180%	ref.^38^
WSe_2_ monolayer	650	2	1.8 × 10^5^	3.5 × 10^5^	ref. 39
Mo_0.5_W_0.5_S_2_ polycrystal film	635	2.2	5.8	11.35%	ref. 40
MoTe_2_	473	0.5	2560	6700	ref. 41
HfSe_2_ multilayer	800	2	3961		ref. 42
In_2_Se_3_ nanosheet	300	5	395	1630	ref. 43
In_2_Se_3_ nanosheet	400	5	110	340	ref. 43
In_2_Se_3_ nanosheet	500	5	59	146	ref. 43
InSe layers	532	5	0.101	0.235	ref. 44
NbSe_2_ nanoflake	532	0.1	2.3	300	ref. 45
NbSe_2_ nanoflake	808	0.1	3.8	300	ref. 45
